# Downregulation of miR-101 contributes to epithelial-mesenchymal transition in cisplatin resistance of NSCLC cells by targeting ROCK2

**DOI:** 10.18632/oncotarget.6852

**Published:** 2016-01-09

**Authors:** Zhiqiang Ye, Shengli Yin, Zhongzhen Su, Mingjun Bai, Haibo Zhang, Ziqing Hei, Songwang Cai

**Affiliations:** ^1^ Department of Emergency, The Third Affiliated Hospital, Sun Yat-sen University, Guangzhou, China; ^2^ Department of Cardiac Surgery, The First Affiliated Hospital, Sun Yat-sen University, Guangzhou, China; ^3^ Department of Ultrasonography, The Third Affiliated Hospital, Sun Yat-sen University, Guangzhou, China; ^4^ Department of Radiology, The Third Affiliated Hospital, Sun Yat-sen University, Guangzhou, China; ^5^ Department of Medical Research Center, The Third Affiliated Hospital, Sun Yat-sen University, Guangzhou, China; ^6^ Department of Anesthesiology, The Third Affiliated Hospital, Sun Yat-sen University, Guangzhou, China; ^7^ Department of Cardiothoracic Surgery, The Third Affiliated Hospital, Sun Yat-sen University, Guangzhou, China

**Keywords:** miR-101, non-small cell lung cancer, epithelial-mesenchymal transition, chemoresistance, ROCK2

## Abstract

Chemoresistance and epithelial-mesenchymal transition (EMT) in cancer are linked phenomena. EMT contributes to chemoresistance, however, little is known about whether chemotherapy can induce EMT in cancer cells. Here, we found that miR-101 expression was downregulated in cisplatin-resistant non-small cell lung cancer (NSCLC) cells. Restoration of miR-101 expression inhibited EMT and increased the sensitivity of cisplatin-resistant NSCLC cells to cisplatin *in vitro* by targeting ROCK2. Furthermore, ROCK2 protein level was inversely correlated with miR-101 level in NSCLC tissue samples. Kaplan-Meier analysis revealed that low miR-101 expression in NSCLC was correlated with poor survival time. In summary, our results provide novel mechanistic insights into the role of miR-101/ROCK2 signaling in the cisplatin resistance of NSCLC cells. Targeting of miR-101 is a potential therapeutic approach for NSCLC.

## INTRODUCTION

Non-small cell lung cancer (NSCLC) is the most common lung cancer and is the leading cause of death from cancer throughout the world [1]. Tumor metastasis is the primary cause of death in NSCLC patients [2]. Although surgical resection and adjuvant therapy can effectively treat NSCLC, patients with tumor metastasis are mostly incurable because of its systemic nature and the resistance of disseminated tumor cells to existing therapeutic agents, including chemotherapy [3]. In cancer, chemoresistance and metastasis are linked phenomena, but the molecular basis for this link is still largely unknown [4]. Epithelial-mesenchymal transition (EMT) in cancer cells plays a critical role in tumor metastasis [5]. Accumulating evidence shows that EMT contributes chemoresistance, however, little is known about whether chemotherapy can induce EMT in cancer cells [6, 7].

MicroRNAs (miRNAs) are a class of endogenously expressed, non-coding RNAs of approximately 22 nucleotides in size. miRNAs regulate gene expression through translational inhibition or cleavage of target mRNA molecules [8]. Emerging evidence has shown that miRNAs play an important role in chemoresistance by modulating EMT [6, 9–12]. miR-101 is a critical tumor suppressor in multiple tumor types, including NSCLC [13–16]. However, little is known about its role in the induction of EMT by chemoresistance.

In this study, we found that miR-101 expression was downregulated in cisplatin-resistant NSCLC cells. Restoration of miR-101 expression inhibited EMT and promoted the sensitivity of cisplatin-resistant NSCLC cells to cisplatin *in vitro* by targeting ROCK2. ROCK2 protein levels were inversely correlated with miR-101 levels in NSCLC tissue samples. Kaplan-Meier analysis revealed that low miR-101 expression in NSCLC was correlated with poor survival time. In conclusion, our results provide novel mechanistic insight into the role of miR-101/ROCK2 signaling in the cisplatin resistance of NSCLC cells. Targeting miR-101 is a potential therapeutic approach for NSCLC.

## RESULTS

### Differences in biological functions of parental A549/A549-res and NCI-H520/NCI-H520-res cells

**Figure 1:**
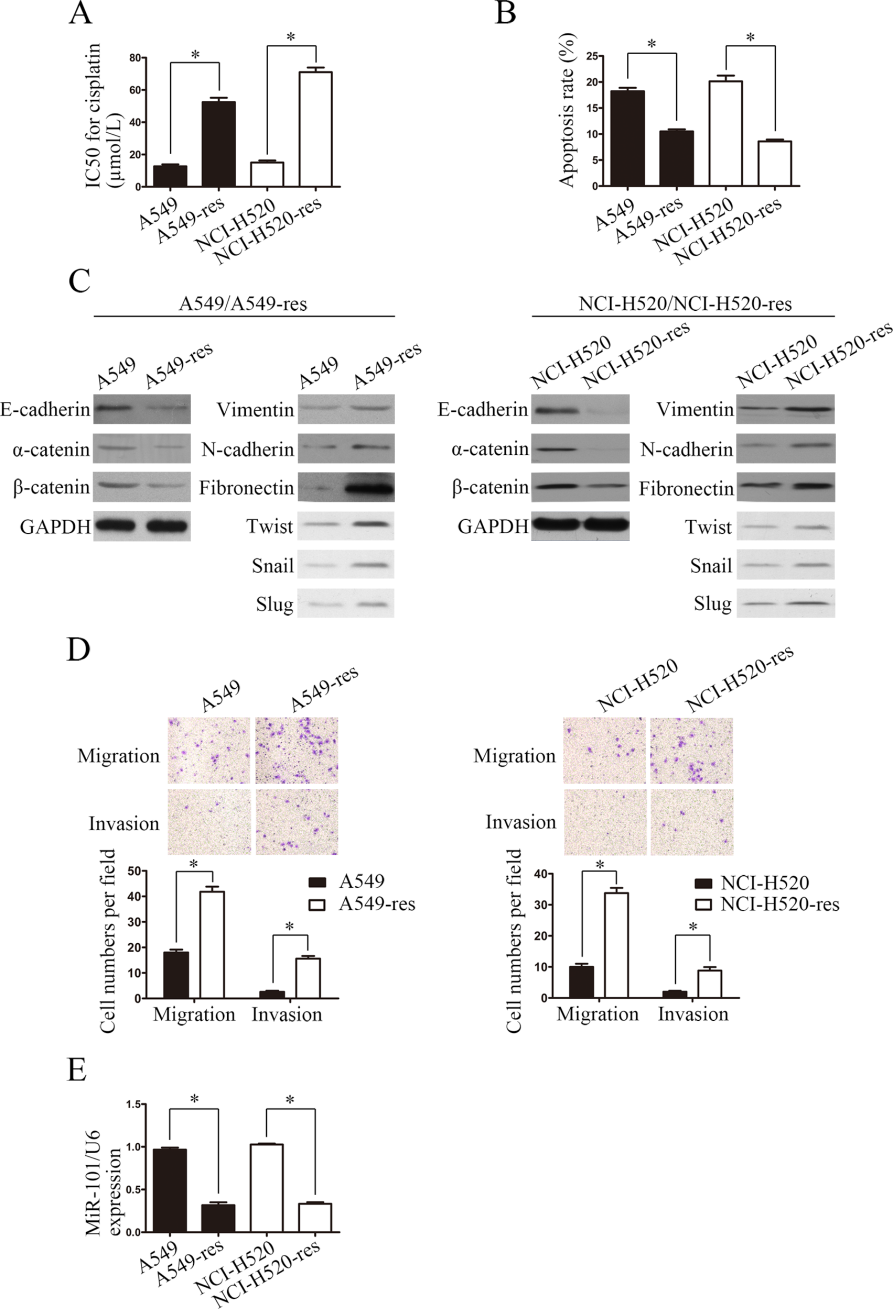
Differences between NSCLC cells and NSCLC-res cells **A.** Cisplatin sensitivity of A549/A549-res and NCI-H520/NCI-H520-res cells was examined by MTT assay. **B.** Flow cytometry analyses of the percentages of apoptotic A549/ A549-res and NCI-H520/NCI-H520-res cells treated with cisplatin. **C.** Expressions of epithelial and mesenchymal markers in A549/A549-res and NCI-520-res cells were measured by western blotting. **D.** Migration and invasion abilities were examined in A549/A549-res and NCI-520-res cells by transwell migration assays (left) and Matrigel invasion assays (right). Magnification ×100. **E.** miR-101 expression in A549/A549-res and NCI-520-res cells was examined by real-time PCR. A549-res, A549 cisplatin resistant; NCI-H520-res, NCI-H520 cisplatin resistant.

To establish cisplatin-resistant NSCLC cells, we maintained A549 and NCI-H520 cells with cisplatin as previously reported [11]. The cisplatin IC50 values of A549-res and NCI-H520-res cells increased by 4.1 and 4.7-fold, respectively, compared with the associated parental lines (Figure 1A). The apoptosis rates of A549-res and NCI-H520-res cells were also lower than those of their respective parental lines (*P* < 0.05) (Figure 1B). Emerging evidence indicates that cisplatin-resistant cancer cells, including NSCLC-res cells, have a mesenchymal phenotype [7, 10, 12–13]. To further explore the mechanism behind this phenotype, we examined the expression of epithelial markers and mesenchymal markers in A549/A549-res and NCI-H520/NCI-H520-res cells. The results showed that, compared with their parental lines, the expressions of mesenchymal markers (vimentin, fibronectin and N-cadherin) increased significantly and the expressions of epithelial markers (E-cadherin, α-catenin and β-catenin) decreased dramatically in A549-res and NCI-H520-res cells (Figure 1C). In addition, transwell migration assays and Matrigel invasion assays showed that the migration and invasion abilities of A549-res and NCI-H520-res cells increased significantly compared with those of their parental lines (Figure 1D). These results indicate that chemoresistant A549-res and NCI-H520-res cells had undergone EMT and had increased migration and invasion abilities.

Our results were similar to those of other studies in showing that miR-101 plays a critical role in NSCLC by inhibiting NSCLC cell proliferation, migration, and invasion and promoting NSCLC cell apoptosis (data no shown) [16, 17–18]. However, the role of miR-101 in NSCLC cell chemoresistance is still largely unknown. Here, we examined miR-101 expression by real-time PCR. The results showed that miR-101 expression was downregulated in A549-res and NCI-H520-res cells compared with A549 and NCI-H520 cells (Figure 1E).

### Restoration of miR-101 expression inhibits EMT and promotes the sensitivity of cisplatin-resistant NSCLC cells to cisplatin *in vitro*

**Figure 2:**
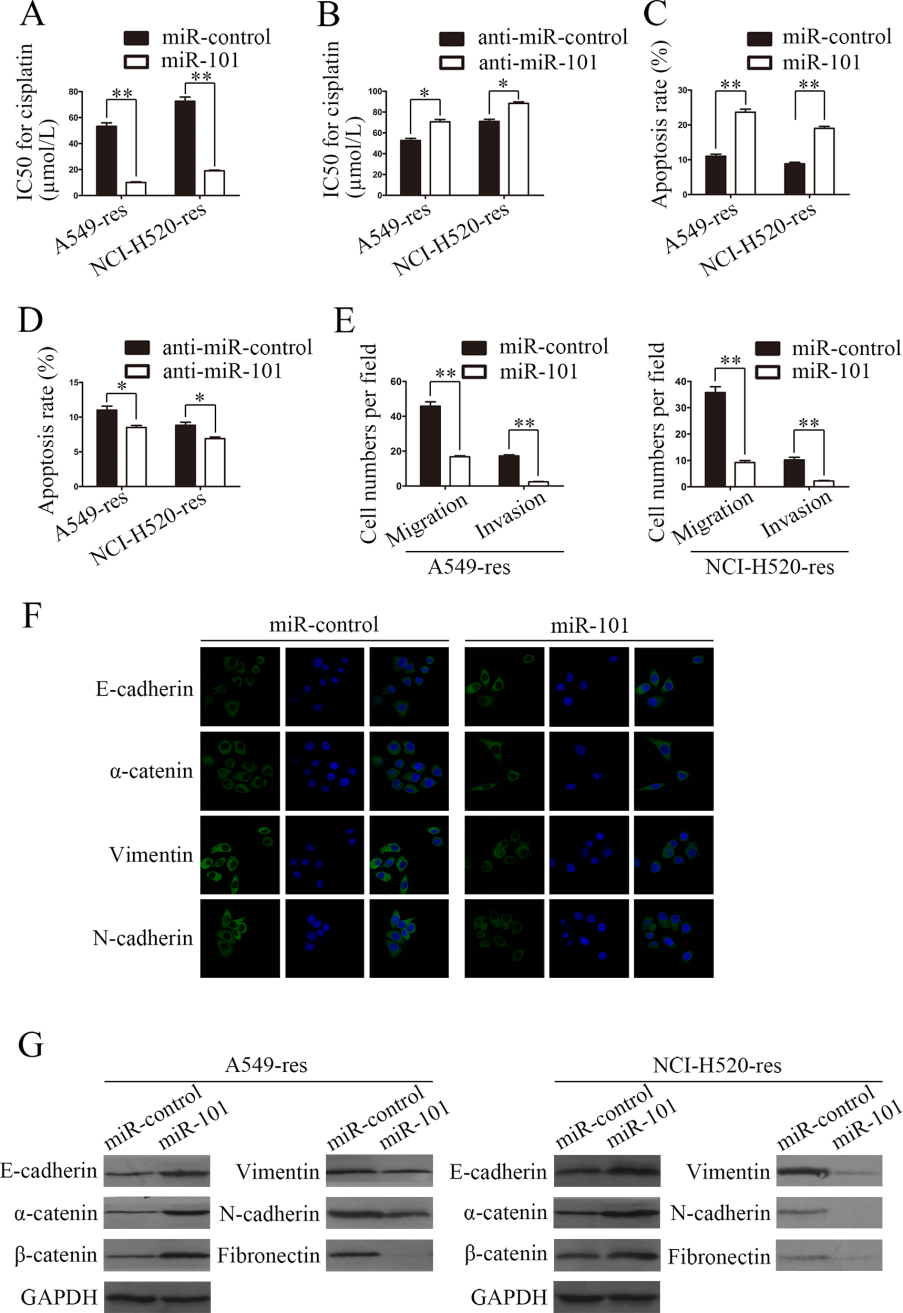
Restoration of miR-101 expression inhibits EMT and promotes the sensitivity of NSCLC cells to cisplatin *in vitro* **A.** The IC50 for cisplatin of A549-res and NCI-H520-res cells transfected with miR-101 mimics was significantly lower than for cells transfected with miR-control. **B.** The cisplatin IC50 values of A549-res and NCI-H520-res cells transfected with anti-miR-101 mimics were higher than for cells transfected with anti-miR-control. **C.** The apoptosis rates of A549-res and NCI-H520-res cells transfected with miR-101 mimics were higher than those of cells transfected with miR-control. **D.** The apoptosis rates of A549-res and NCI-H520-res cells transfected with anti-miR-101 mimics were lower than those of cells transfected with anti-miR-control. **E.** Transwell migration assays and Matrigel invasion assays showed that restoration of miR-101 expression inhibited migration and invasion abilities of A549-res and NCI-H520-res cells and that anti-miR-101 overexpression promoted those abilities. **F.** Immunofluorescence assays in A549-res cells revealed that miR-101 overexpression dramatically promoted the expression of epithelial markers (E-cadherin and α-catenin) and dramatically suppressed the expression of mesenchymal markers (vimentin and N-cadherin). **G.** Western blotting assays showed that restoration of miR-101 expression significantly increased the expressions of epithelial markers and significantly inhibited the expressions of mesenchymal markers.

To explore whether miR-101 is involved in the mechanism of chemotherapy-induced EMT, chemoresistant A549-res and NCI-H520-res cells were transfected with miR-101 or anti-miR-101 mimics, respectively. The cisplatin IC50 values for A549-res and NCI-H520-res cells transfected with miR-101 mimics were 81.3% and 73.9% lower than for A549-res and NCI-H520-res cells transfected with miR-control, respectively (Figure 2A), while the cisplatin IC50 values of A549-res and NCI-H520-res cells transfected with anti-miR-101 mimics were 1.3-fold and 1.2-fold higher than for A549-res and NCI-H520-res cells transfected with anti-miR-control, respectively (Figure 2B). Furthermore, the apoptosis rates of A549-res and NCI-H520-res cells were increased significantly via transfection with miR-101 mimics (Figure 2C) and decreased dramatically by transfection with anti-miR-101 mimics (Figure 2D).

Transwell migration assays and Matrigel invasion assays showed that restoration of miR-101 expression inhibited the migration and invasion abilities of A549-res and NCI-H520-res cells and that anti-miR-101 overexpression promoted those abilities (Figure 2E). All these results indicate that miR-101 overexpression promotes the sensitivity of NSCLC cells to cisplatin *in vitro*.

To explore whether miR-101 overexpression inhibits EMT, we performed immunofluorescence assays in A549-res cells, and the results revealed that miR-101 overexpression dramatically promoted the expression of epithelial markers (E-cadherin and α-catenin) and dramatically suppressed the expression of mesenchymal markers (vimentin and N-cadherin) (Figure 2F). Furthermore, western blotting assays were carried out using A549-res and NCI-H520-res cells, and the results showed that restoration of miR-101 expression significantly increased the expression of epithelial markers (E-cadherin, α-catenin and β-catenin) and significantly inhibited the expression of mesenchymal markers (vimentin, fibronectin and N-cadherin) (Figure 2G). These results suggest that low miR-101 expression is responsible for chemotherapy-induced EMT in NSCLC cells.

### ROCK2 is a direct regulatory target of miR-101

**Figure 3:**
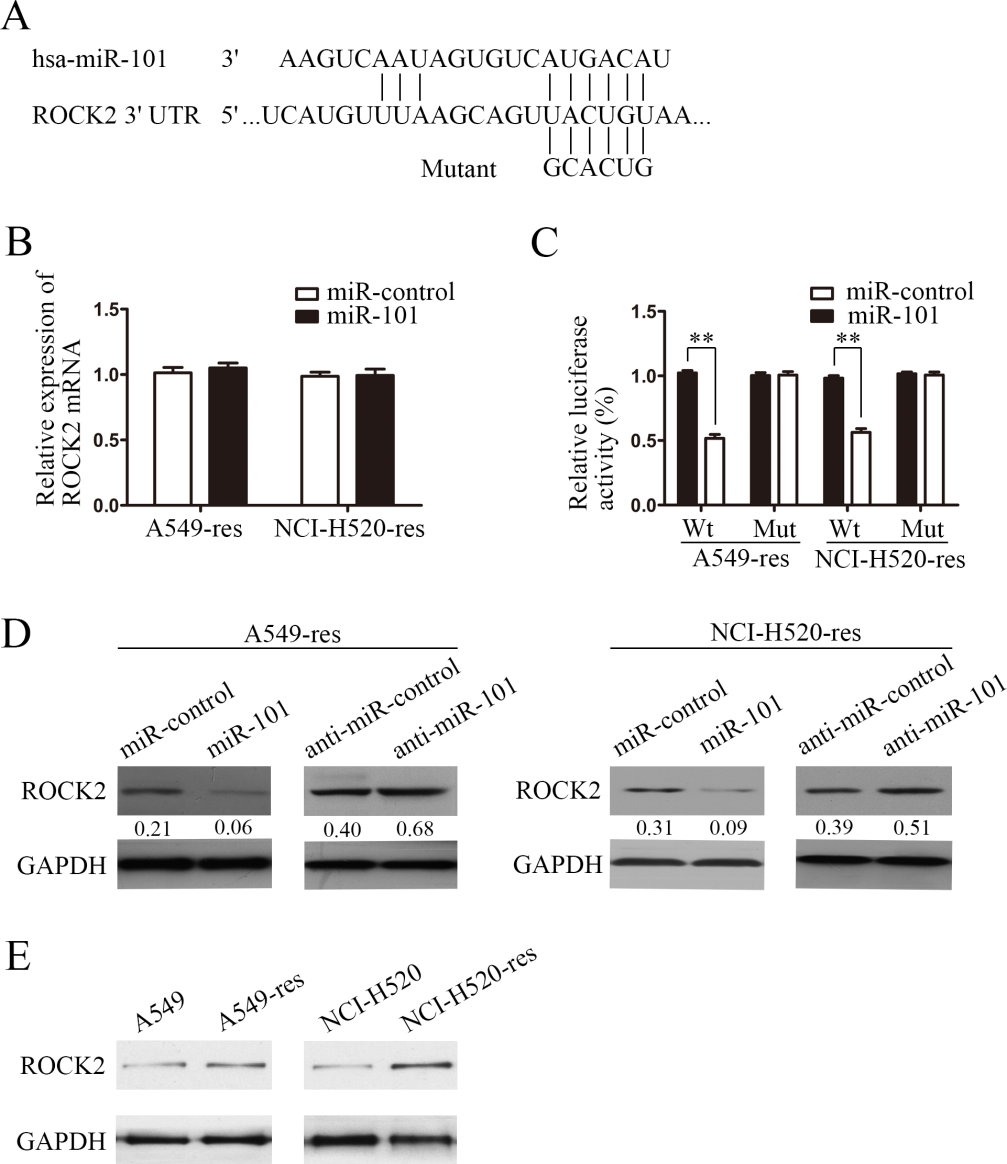
ROCK2 is a direct target of miR-101 **A.** Schematic of predicted miR-101 binding sequence in ROCK2 3′-UTR. ROCK2 3′-UTR was mutated at the site complementary to the seed region of miR-101, as indicated. Human ROCK2 3′-UTR fragments containing a wild-type or mutant miR-101 binding sequence were cloned downstream of the luciferase reporter gene. **B.** Real-time PCR of ROCK2 in A549 and NCI-H520 cells transfected with miR-101 mimics or miR-control. Data were normalized to GAPDH mRNA expression. **C.** Luciferase activity of wild-type (Wt) and mutant (Mut) ROCK2 3′-UTR gene in A549 and NCI-H520 cells transfected with miR-101 mimics or miR-control. **D.** ROCK2 immunoblotting in A549 and NCI-H520 cells transfected with miR-101 mimics and miR-control or with anti-miR-101 and anti-miR-control. **E.** ROCK2 immunoblotting in A549-res and NCI-H520-res cells compared with the associated parental lines.

To explore how downregulation of miR-101 contributes to chemotherapy-induced EMT, we used a prediction program (TangetScan) to predict the targets of miR-101. The 3′-UTR of ROCK2 mRNA contains a complementary binding sequence for the seed region of miR-101 (Figure 3A). Restoration of miR-101 expression did not cause degradation of ROCK2 mRNA (Figure 3B); however, it reduced the activity of a luciferase reporter gene fused to a wild-type ROCK2 3′-UTR fragment (*P* < 0.01, Figure 3C), indicating that miR-101 targets ROCK2 through translational inhibition. The effects of miR-101 on the endogenous expression of ROCK2 were further examined by western blotting (Figure 3D). Restoration of overexpression of miR-101 in NSCLC cells resulted in a marked decrease in ROCK2 expression (over fivefold reduction in both A549-res and NCI-H520-res cells), whereas miR-101 inhibitor oligonucleotides induced a pronounced increase in ROCK2 expression. Furthermore, we examined the levels of ROCK2 using western blotting and the results demonstrated that the levels of ROCK2 were upregulated in A549-res and NCI-H520-res cells compared with their parental cells (Figure 3E), which were inversely correlated with the level of miR-101. These data suggest that miR-101 inhibited ROCK2 expression at the post-transcriptional level by directly targeting the 3′-UTR of ROCK2 mRNA.

### ROCK2 is involved in miR-101-induced EMT and cisplatin resistance

**Figure 4:**
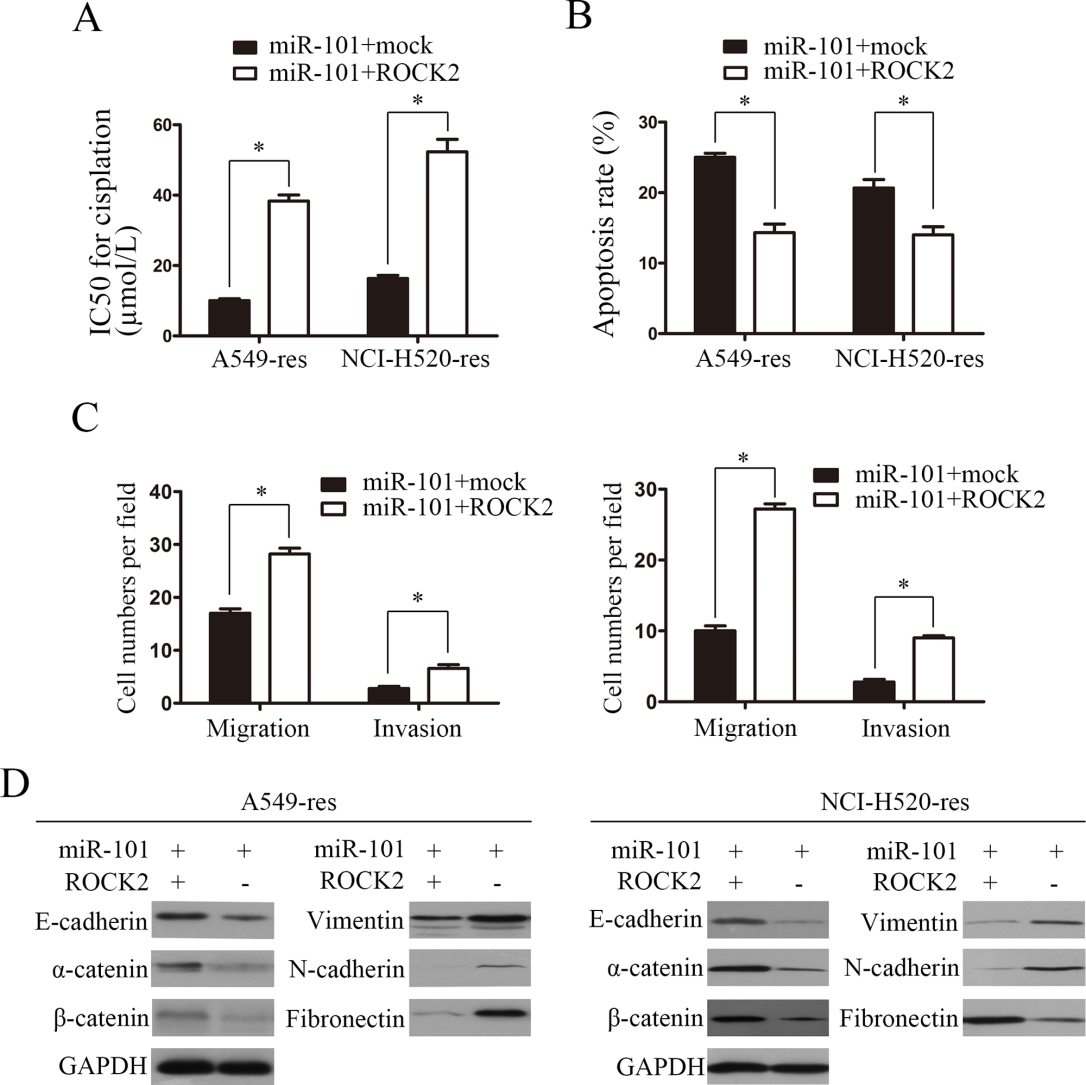
ROCK2 is involved in miR-101-induced EMT and cisplatin resistance **A.** The cisplatin IC50 values of A549-res and NCI-H520-res cells co-transfected with miR-101 mimics and ROCK2 were significantly higher than those of cells transfected with miR-101 and mock. **B.** The apoptosis rates of A549-res and NCI-H520-res cells co-transfected with miR-101 mimics and ROCK were remarkably lower than those of cells transfected with miR-101 and mock. **C.** Effects of miR-101 mimics on invasion and migration of A549-res and NCI-H520-res cells were rescued by overexpressing ROCK2. **D.** ROCK2 overexpression reversed the miR-101-mediated inhibition of the expression of epithelial markers (E-cadherin, α-catenin and β-catenin) and the promotion of the expression of mesenchymal markers (vimentin, fibronectin and N-cadherin).

To explore whetherROCK2 was involved in miR-101-induced EMT and cisplatin resistance in NSCLC cells, we performed rescue experiments by co-transfecting A549-res and NCI-H520-res cells with miR-101 mimics and a ROCK2 plasmid or mock plasmid. The results of the cell viability assays showed that the cisplatin IC50 values of A549-res and NCI-H520-res cells transfected with ROCK2 were significantly increased compared with the control group (Figure 4A). The results of the apoptosis assays showed that restoration of ROCK2 expression significantly decreased the percentage of cisplatin-induced apoptotic cells (Figure 4B). In addition, the transwell migration assays and Matrigel invasion assays showed that ROCK2 overexpression reversed the miR-101-mediated inhibition of migration and invasion in A549-res and NCI-H520-res cells (Figure 4C). All these results indicate that ROCK2 overexpression can reverse cisplatin **s**ensitization mediated by miR-101 overexpression in NSCLC cells. Furthermore, western blotting assays were performed and showed that ROCK2 overexpression reversed the miR-101-mediated inhibition of the expression of epithelial markers (E-cadherin, α-catenin and β-catenin) and the miR-101-mediated promotion of the expression of mesenchymal markers (vimentin, fibronectin and N-cadherin) (Figure 4D).

### ROCK2 protein levels were inversely correlated with miR-101 levels in NSCLC tissue samples

**Figure 5:**
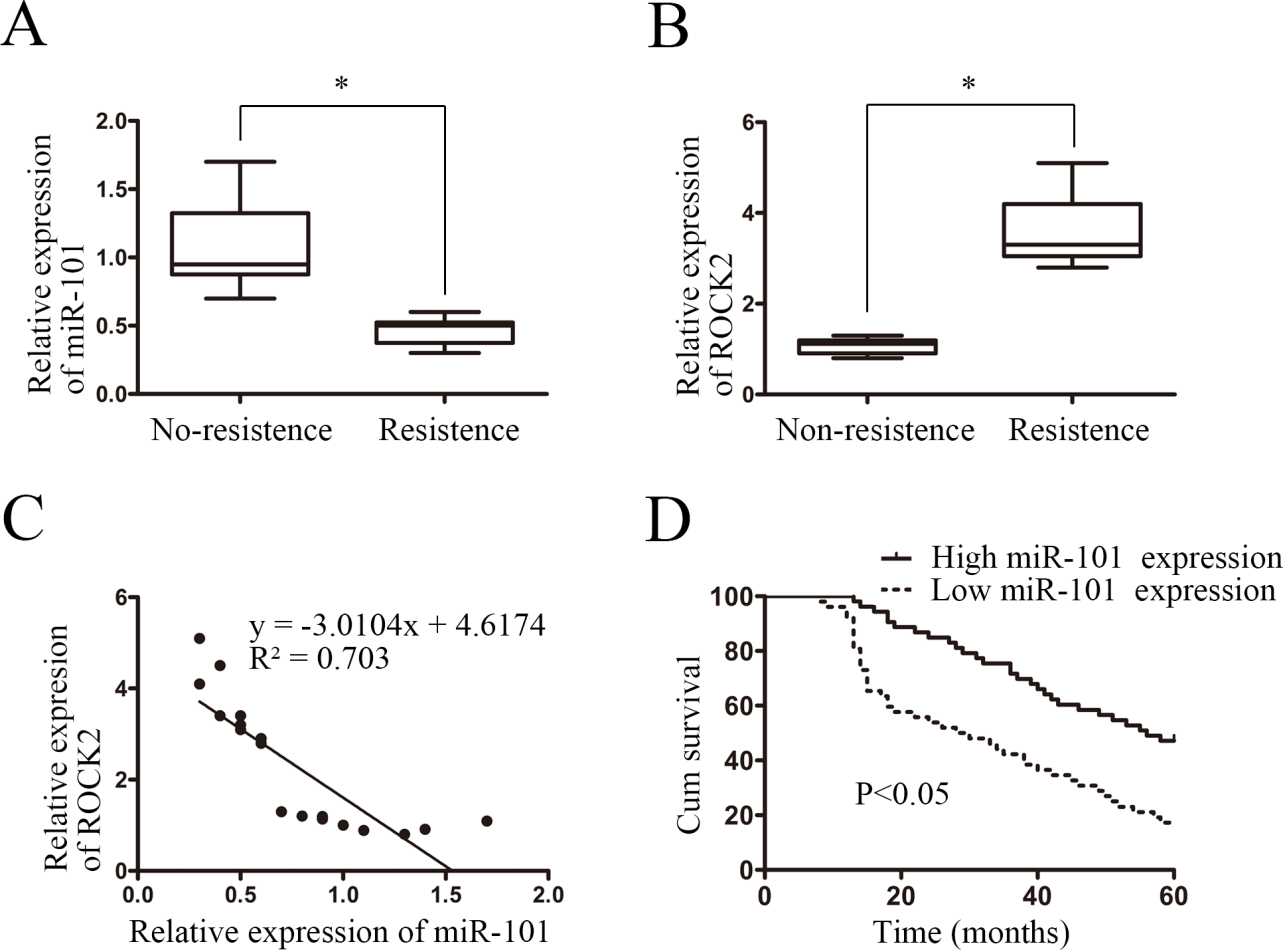
ROCK2 protein levels were inversely correlated with miR-101 levels in NSCLC tissue samples **A.** Real-time PCR showed that 10 chemoresistant NSCLC tissue samples had lower miR-101 expression than 10 non-chemoresistant NSCLC tissue samples (*P* < 0.05). **B.** Real-time PCR showed that ROCK2 mRNA levels were significantly upregulated in 10 chemoresistant NSCLC tissue samples compared with 10 non-chemoresistant NSCLC tissue samples (*P* < 0.05). **C.** ROCK2 upregulation was inversely correlated with the degree of miR-101 downregulation in the 10 chemoresistant NSCLC and 10 non-chemoresistant NSCLC tissue samples (R^2^ = 0.703, *P* < 0.05). **D.** Kaplan-Meier analysis of survival times of patients with NSCLC as a function of miR-101 levels.

To further explore whether the biological effects of the downregulation of miR-101 were correlated with ROCK2 mRNA levels in clinical NSCLC tissue samples, miR-101 expression and ROCK2 mRNA levels were examined in 10 chemoresistant NSCLC tissue samples and 10 non-chemoresistant NSCLC tissue samples by real-time PCR. The results showed that the 10 chemoresistant NSCLC tissue samples had lower miR-101 expression than the 10 non-chemoresistant NSCLC tissue samples (*P* < 0.05) (Figure 5A). Conversely, ROCK2 mRNA levels were significantly upregulated in the chemoresistant NSCLC tissue samples (*P* < 0.05) (Figure 5B). The extent of ROCK2 upregulation was inversely correlated with the degree of miR-101 downregulation (R^2^ = 0.703, *P* < 0.05) (Figure 5C), suggesting that the inhibitory effects of miR-101 on ROCK2 were clinically relevant in NSCLC.

To explore the prognostic significance of miR-101 expression in patients with NSCLC, the expression of miR-101 in a cohort of 105 NSCLC tissue samples was determined by real-time PCR. The median miR-101 expression level of all 105 NSCLC tissue samples was chosen as the cut-off point for separating tumors with low miR-101 expression compared with those with high miR-101 expression. The data showed that 52 and 53 of 105 NSCLC tissue samples exhibited low and high miR-101 expression, respectively (Table 1). The correlation analysis revealed that low miR-101 expression was associated with differentiation, lymph node metastasis, distant metastasis, and clinical stage. These results suggested that low miR-101 expression was correlated with NSCLC progression. Kaplan-Meier analysis showed that low miR-101 expression in NSCLC was correlated with poor survival time (*P* < 0.05, Figure 5D). Furthermore, multivariate Cox regression analysis suggested that low miR-101 expression was an independent prognostic factor for poor survival in patients with NSCLC (*P* = 0.038, Table 2).

**Table 1:** Correlation of miR-101 expression in tissues with clinicopathological variables of patients in 105 cases of non-small cell lung cancer

Variables	miR-101			P Value^*^
	All cases (*n* = 105)	High expression (*n* = 53)	Low expression (*n* = 52)	
Age				
≤60	47	22	25	0.499
>60	58	31	27	
Gender				
Male	67	32	35	0.460
Female	38	21	17	
Histological type				
SqCC	29	12	17	0.335
A	64	36	28	
Other	12	5	7	
Differentiation				
Low	60	23	37	0.004
Moderate+ high	45	30	15	
T factor				
T1 + T2	55	33	22	0.041
T3+T4	50	20	30	
Lymph node				
N0 + N1	60	36	24	0.024
N2 + N3	45	17	28	
Distant metastasis				
M0	97	52	45	0.031
M1	8	1	7	
Clinical stage				
I+II	54	36	18	0.001
III+IV	51	17	34	

* χ^2^ tests, SqCC, squamous cell cancer.

**Table 2:** Univariate and multivariate analysis of factors associated with overall survival time of patients with nonsmall cell lung cancer

Clinical variable	Case number	HR (95% CI)	P Value
**Univariate analysis**			
miR-101 (Low vs High)	52/53	2.467 (1.528-3.984)	< 0.001
Age (>60 vs ≤60)	58/47	0.896 (0.561-1.430)	0.646
Gender (Female vs Male)	38/67	0.989 (0.610-1.603)	0.963
Histological type (SqCC vs A vs Other)	29/64/12	1.179 (0.795-1.749)	0.413
Differentiation (Moderate+high vs Low)	45/60	0.600 (0.370-0.974)	0.039
Distant metastasis (M1 vs M0)	8/97	9.939 (4.515-21.879)	< 0.001
T factor (T3+T4 VS T1+T2)	50/55	1.722 (1.073-2.761)	0.024
Lymph node (N2+N3 vs N0+N1)	45/60	6.612 (3.897-11.219)	< 0.001
Clinical stage (III + IV vs I + II)	51/54	9.842 (5.516-17.560)	< 0.001
**multivariate analysis**			
miR-101 (Low vs High)	52/53	1.780 (1.033-3.067)	0.038
Differentiation (Moderate+high vs Low)	45/60	0.834 (0.491-1.415)	0.501
T factor (T3 + T4 vs T1 + T2)	50/55	1.051(0.625-1.768)	0.852
Lymph node (N2 + N3 vs N0 + N1)	45/60	0.519 (0.206-1.311)	0.165
Clinical stage (III + IV vs I + II)	51/54	14.798 (5.113-42.835)	< 0.001

## DISCUSSION

Here, we showed the functional role of low miR-101 expression in promoting EMT in cisplatin-resistant NSCLC cells. This mechanistic research showed that ROCK2 was the direct target of miR-101 and that ROCK2 overexpression reversed miR-101-mediatedEMT and cisplatin resistance in NSCLC cells. Furthermore, this research indicated that ROCK2 protein levels were inversely correlated with miR-101 levels in NSCLC tissue samples and that low miR-101 expression was correlated with poor survival time.

EMT is a primary embryonic process during which polarized epithelial cells become motile mesenchymal cells [19]. EMT is closely associated with cancer progression [20], as it is involved in three major steps of malignant cancer progression: invasion, dissemination and metastasis [3]. Cancer therapy, including chemotherapy, often results in acquired resistance. It is widely accepted that tumors undergoing EMT may resist conventional chemotherapy [5, 21]. The possible mechanism may be that the induction of EMT in normal epithelial cell populations generates cancer stem cells, which have increased resistance to chemotherapy [22–23]. To better explore the mechanism of chemoresistance, it is very important to know how to induce cancer cell undergoing EMT. Several phenomena correlated with tumor initiation, such as inflammation, physical constraints and metabolic stress, are known to trigger the expression of EMT-promoting factors [19]. Interestingly, here we showed that chemoresistance may help to introduce EMT in NSCLC, not only EMT contributed to chemoresistance. Recent study also shown that chemoresistance may promote cancer cells to undergo EMT with the acquisition of EMT markers and the loss of the epithelial phenotype [5]. Subsequently, we investigated the possible mechanism.

MiR-101 has been implicated as a critical tumor suppressor miRNA in different types of human cancers, including lung cancer [13, 17, 18, 24]. Reduced expression of miR-101 in patients with NSCLC has been shown to be related to reduced overall survival [25] through the promotion of cell proliferation, tumorigenesis, and invasion. Previous studies have indicated that low miR-101 expression contributes to chemotherapy-resistance in epithelial ovarian cancer [26], bladder cancer [27] and lung cancer [16]. However, whether miR-101 plays an important role in chemotherapy-induced EMT is unclear. In this study, we found that chemotherapy-resistant NSCLC cells had a lower level of miR-101 expression compared with normal NSCLC cells. Furthermore, miR-101 bound the complementary sites in the 3′-UTR of Rho-associated coiled-coil containing protein kinase 2 (ROCK2) and significantly reduced ROCK2 protein expression. MiR-101 plays an important role in chemotherapy-induced EMT by directly targeting the EMT inducer ROCK2.

ROCK2 is a serine/threonine kinase that regulates cytokinesis, smooth muscle contraction, the formation of actin stress fibers and focal adhesions. ROCK2 functions as a key downstream effector of RhoA small GTPase to play a critical role in the oncogenesis of prostate, bladder, fibrosarcoma, melanoma, liver and lung cancer [28–29]. Little is known about the role of ROCK2 in chemotherapy-induced EMT in lung cancer. Whether ROCK2 is the target gene of miR-101 is still unclear. In this study, our data suggested that high expression of ROCK2, which is a target of miR-101, contributes to cisplatin resistance in NSCLC cells by inducing epithelial-mesenchymal transition.

In conclusion, we found that downregulation of miR-101 contributes to epithelial-mesenchymal transition in cisplatin-resistant NSCLC cells by targeting ROCK2. Targeting miR-101 is a potential therapeutic approach for cisplatin-resistant NSCLC.

## MATERIALS AND METHODS

### Tissue specimens

NSCLC tissues and adjacent normal tissues were acquired after obtaining informed consent from patients at the Third Affiliated Hospital, Sun Yat-sen University (Guangzhou, China) between January 2003 and January 2008. All patients' diagnoses were confirmed histopathologically. This study was approved by the institutional research ethics committee.

### Cell lines and cell culture

Human bronchial epithelial cells (HBE) and NSCLC cells (A549, NCI-520, NCI-460, NCI-H596) were used in this study. The cell lines were purchased from Cell Bank, Chinese Academy of Sciences (Shanghai, China). The NSCLC cell lines were cultured in RPMI 1640 (Gibco, Invitrogen Life Technologies, Carlsbad, USA) supplemented with 10% newborn calf serum (Gibco, Invitrogen Life Technologies, Carlsbad, USA), and HBE cells were maintained in keratinocyte serum–free medium with bovine pituitary extract and recombinant epidermal growth factor (Invitrogen Life Technologies, Carlsbad, USA). Cells were transfected with DNA constructs using siPORT™ NeoFX™ Transfection Agent (Ambion, USA).

### RNA isolation and quantitative real-time PCR

TRIzol reagent (Invitrogen Life Technologies, Carlsbad, USA) was used to extract RNA. cDNA was synthesized with the PrimeScript RT Reagent Kit (Promega, Madison, WI). Real-time PCR was carried out using the ABI 7900HT Fast Real-Time PCR system (Applied Biosystems, CA, USA).

### Vector construction

The pre-miR-101 and pre-miR-101-sponge-inhibitor sequences and the ROCK2 plasmid were synthesized by GenePharma (Shanghai, China).

### Immunofluorescence

A549 cells were cultured on cover glasses, fixed using 4% paraformaldehyde, and permeabilized with 0.1% Triton X-100 in TBS. The cover glasses were incubated with the primary antibodies (E-cadherin, α-catenin, vimentin and N-cadherin, Santa Cruz Biotechnology) at 1:50 dilutions. E-cadherin, α-catenin, vimentin and N-cadherin were detected with an anti-goat secondary antibody conjugated to Alexa Fluor 488 (Invitrogen Life Technologies). The fluorescent staining was visualized using a 63× NA 1.3 oil objective on a confocal microscope (LSM 510 Meta; Carl Zeiss, Inc.). The images were captured with sequential acquisition settings at a resolution of 512 × 512 pixels with a 12-bit depth. The confocal settings were fixed for the duration of the experiments to enable the comparisons of the fluorescence intensities.

### Luciferase reporter assay

Luciferase reporter assays were performed as described previously [30]. Briefly, the putative miR-101 binding sequence of ROCK2 and a mutated sequence of the 3′-UTR of ROCK2 were cloned and named Wt ROCK2 3′UTR and Mut ROCK2 3′UTR. Cells grown in a 48-well plate were infected with wild-type or mutated reporter plasmid and miR-101 or miR-control. A dual luciferase assay was performed 48 h after infection. Luciferase activity was measured using the Dual-Luciferase Reporter Assay System (Promega).

### Western blotting

The following primary antibodies were used: anti-ROCK2 antibody (1:1000; Santa Cruz Biotechnology) and anti–glyceraldehyde-3-phosphate dehydrogenase (GAPDH) antibody (1:8000, Cell Signaling). GeneTools software (version 3.03; Syngene, Cambridge, UK) was used to measure the intensities of the protein bands.

### Cell viability assay

Cells were seeded into 96-well plates at a density of 2 × 10^3^ cells per well. Twenty-four hours later, cisplatin was added to the cells at different final concentrations. Forty-eight hours later, cell viability was assessed by MTS assay (Promega).

### Apoptosis assay

Twenty-four hours after transfection, cisplatin (60 μM) was added to the cell culture medium for 48 h, and then the cells were collected for analysis. Apoptosis was examined using an Annexin VFITC/ PI Apoptosis Detection Kit (KeyGEN, China) according to the manufacturer's protocol.

### Matrigel invasion assays and transwell migration assays

For the Matrigel invasion assays, cells (5 × 10^4^) were plated in a Matrigel invasion chamber (BD Biosciences) without serum; serum was used as a chemoattractant. Twenty-four h later, the non-invading cells were removed with cotton swabs. Migrated and invaded cells located on the lower side of the chamber were fixed in formaldehyde and then stained with crystal violet. The transwell migration assays were carried out in a similar manner but without Matrigel on the top side of the filter.

### Statistical analysis

Statistical analyses were carried out using SPSS software (version 17.0). The χ2 test was used to assess differences between variables. Kaplan-Meier analysis was used for the survival analysis. Differences in overall survival were analyzed by the log-rank test. P < 0.05 was considered to indicate a statistically significant difference.
